# Seizure freedom in epilepsia partialis continua (EPC) through vagus nerve stimulation (VNS) therapy: A case report^☆^

**DOI:** 10.1016/j.ebcr.2013.01.002

**Published:** 2013-04-03

**Authors:** Yuqin Shen, Feng Xia, Guodong Feng, Lijuan Liu, Wei Lin, Yonghong Liu, Ming Shi, Xianhui Ren, Bojun Ding, Gang Zhao, Yanchun Deng

**Affiliations:** a Department of Neurology, Xijing Hospital, Fourth Military Medical University, Xi'an 710032, China; b Department of Neurosurgery, Xijing Hospital, Fourth Military Medical University, Xi'an 710032, China

**Keywords:** Epilepsia partialis continua, VNS

## Abstract

Vagus nerve stimulation (VNS) is generally considered as a palliative treatment for patients with drug-resistant partial-onset epilepsy. We report a case in which a patient with drug-resistant epilepsia partialis continua (EPC), became seizure-free for 15 months with VNS combined with antiepileptic medication regimens. To our knowledge, similar cases have not been reported previously.

## 1 Introduction

Vagus nerve stimulation (VNS) is an effective adjunctive therapy for patients with drug-resistant partial-onset epilepsy [1]. It decreases seizure frequency by approximately 50% in 30–40% of implanted patients [2]. The efficacy of VNS has also been demonstrated in treating various types of generalized epilepsies, including genetic generalized epilepsy (GGE) and Lennox–Gastaut syndrome [3]. Here, we report that a patient became seizure-free in epilepsia partialis continua (EPC) with VNS therapy combined with antiepileptic drug (AED) regimens.

## 2 Case report

**Fig. 1:**
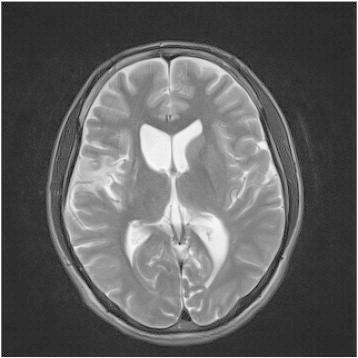
The signal of the cortex in the head of the right putamen has disappeared, the lenticular nucleus is somewhat small, and the right frontotemporal lobe and insular lobe show an abnormal signal, with suspected developmental delay.

A 21-year-old, right-handed man started having seizures at the age of 18. He reported countless limb shaking for about 10 s and occasional generalized tonic-clonic seizures at a frequency of 5–7 times per year. His seizures were commonly precipitated by stress, sleep deprivation, AED noncompliance, and changes in emotion, such as excitation and anger. He was initially treated with valproic acid (VPA) with minimal benefits. He was also treated with carbamazepine (CBZ), which provided better seizure control, but its effectiveness disappeared four months later. Then, he was treated with lamotrigine (LTG) at 200 mg daily, levetiracetam (LEV) at 1250 mg daily, and topiramate (TPM) at 200 mg daily with minimal benefits for the last several months. His general physical and neurological examinations were normal, and his family history was noncontributory. However, he suffered head trauma at the age of three, and his brain MRI revealed that the signal of the cortex in the head of the right putamen disappeared, and the lenticular nucleus was somewhat small, with suspected developmental delay (Fig. 1), and long-term video-EEG monitoring revealed small spike-wave discharges. Sometimes, he could not even say a word or have dinner due to mouth twitching, which also prevented him from working. He was referred by his neurologist to our epilepsy center for evaluation for possible VNS therapy.

A vagus nerve stimulator was subsequently implanted in Sept. 2011. The initial stimulation parameters were the following: current output of 0.25 mA, frequency of 30 Hz, pulse width of 250 μs, 30 s of signal-on time, and 5 min of signal-off time. The patient had two prominent twitches of the mouth per week. Then, his stimulation current output was changed to 0.50 mA, and his seizures became countless as they were pre-operation. Fortunately, he became seizure-free after his stimulation current was reverted to 0.25 mA. After one month of seizure freedom, the patient withdrew from his LEV and TPM medications. He has remained seizure-free for almost 9 months, during which time he has been gainfully employed.

## 3 Discussion

In this particular patient, a history of AED noncompliance may have falsely created the appearance of drug-resistant seizures. In general, patients with VNS rarely achieve seizure freedom [4]. Only one of 198 (0.5%) patients became seizure-free in an early randomized active-control trial. In more recent uncontrolled case studies, significantly higher seizure freedom rates in patients with VNS have been observed. Janszky et al. [5] reported that 6 of 47 (13%) patients became seizure-free, while Ghaemi et al. [6] reported that 10 of 144 (6.9%) patients became free from seizures. Earlier treatment with VNS in the course of a seizure disorder is associated with a greater likelihood of improvement [7]. The patient in this study not only has been seizure-free for 9 months, but was also able to taper off AED treatment VNS implantation. We believe that his remission was achieved by VNS.

Just as any “electronic medication”, the parameter adjustment of VNS, like the dosage of medications, is based on patient profiles. It is a double-edged sword in terms of VNS efficacy for drug-resistant epilepsy. When the standard parameters used are 0.25 mA, 20 Hz, 250 μs, 30 s on, and 5 min off, continuously, the patient experienced two prominent twitches of the mouth weekly. When the parameters were changed (0.50 mA, 20 Hz, 250 μs, 30 s on, and 5 min off, continuously), his seizures became countless as they were pre-operation. When the parameters were adjusted back to 0.25 mA, 20 Hz, 250 μs, 30 s on, and 5 min off, continuously, the patient became seizure-free.

Neurostimulation is a rapidly developing therapy for drug-resistant epilepsy. We believe that it is clinically important to document such cases in the hope of appreciating the clinical features of patients who have the potential to become seizure-free with VNS therapy combined with antiepileptic medication regimens, and, for some patients, the chance to withdraw from the use of AEDs.

## Conflict of interest

There are no conflicts of interest reported by the authors.
